# Does Monopolar Electrocautery Affect the Fetus during Cesarean Section?

**DOI:** 10.3390/medicina60091453

**Published:** 2024-09-05

**Authors:** Sevki Goksun Gokulu, Hamza Yildiz, Ali Yildizbakan, Gorkem Ulger, Huseyin Durukan, Yalcin Celik, Hakan Aytan

**Affiliations:** 1 Department of Obstetrics and Gynecology, Mersin University Faculty of Medicine, 33343 Mersin, Turkey; sevkigoksungokulu@mersin.edu.tr (S.G.G.); hamzayildiz@mersin.edu.tr (H.Y.); aliyildizbakan@mersin.edu.tr (A.Y.); huseyindurukan@mersin.edu.tr (H.D.); drhakanaytan@mersin.edu.tr (H.A.); 2 Department of Neonatology, Mersin University Faculty of Medicine, 33343 Mersin, Turkey; ycelik@mersin.edu.tr

**Keywords:** cesarean section, electrocautery, fetus, newborns, safety

## Abstract

*Background and Objectives:* This study aimed to assess the impact of monopolar electrocautery on the fetus during cesarean section. *Materials and methods:* A retrospective analysis was conducted with 552 patients delivered by cesarean section. Patients were grouped based on usage of monopolar electrocautery. In 272 patients, monopolar electrocautery was used to separate the tissues before the delivery. In 280 patients, no electrocautery was used. Newborn vital signs, Apgar scores, umbilical cord blood parameters, newborn serum parameters collected within 6th postpartum hour, and rate of newborn intensive care unit admission were compared. *Results:* The 1st and 5th minute Apgar scores were significantly higher in the electrocautery group; however, this difference lost its significance at the 10th minute. The median newborn pulse rate (148 (7) vs. 146 (6) beats per minute, *p* = 0.026), umbilical cord blood pH, and partial oxygen pressure were significantly higher in the electrocautery group compared to the no-electrocautery group (7.34 ± 0.06 vs. 7.31 ± 0.06, *p* < 0.001, and 25.5 (14.77) vs. 23 (16.08) mmHg, *p* = 0.025, respectively). The median umbilical cord blood serum calcium level was 1.51 (0.64) mmol/L in the electrocautery group, which was significantly lower than 1.9 (0.82) mmol/L in the no-electrocautery group (*p* = 0.002). The incidence of hypoglycemia was significantly lower in the electrocautery group than in the no-electrocautery group (2.2% vs. 5.7%, *p* = 0.035). *Conclusions:* Monopolar electrocautery during cesarean section affects the fetus, but it is safe to use it. Electrocautery is independently associated with umbilical cord blood pH and calcium level. Electrocautery may be associated with a lower incidence of hypoglycemia.

## 1. Introduction

Electrocautery uses high-frequency alternating current to generate heat in living tissues to make surgical incisions or induce hemostasis. The application of high-frequency alternating electrical current to the tissue results in the occurrence of fulguration, desiccation/coagulation, or vaporization/ablation effects while ensuring the absence of electrical shock. This process aids in tissue division and promotes hemostasis [1].

It has been a crucial component of contemporary surgical practice ever since W. Bovie introduced it and H. Cushing used it for the first time in 1926 and is frequently used in various clinical specialties for surgical procedures, including obstetrics [2,3,4,5]. While electrical cauterization of tissues can improve hemostasis compared to other methods, the heat energy produced during cauterization may diffuse to neighboring tissues and cause significant inflammation and harm [6]. Many worry that utilizing electrosurgical tools can result in excessive scarring and poor wound healing since they cause a thermal burn. Due to growing concerns about surgical site infections, the National Institute for Health and Clinical Excellence produced a guideline in 2008 opposing the use of electrocautery for skin incisions [7]. Numerous research studies have evaluated the effectiveness and safety of electrocautery concerning wound consequences like scarring, infection, separation, and cosmetic features. These studies demonstrated that surgical incisions made with cutting electrocautery can be performed more quickly, with less blood loss, and with fewer postoperative pain complaints than those made with a scalpel, with no statistically significant differences in postoperative wound complication rates, hospital stay length, or wound aesthetic characteristics [8,9].

Obstetrics is a specialization unlike any other because it involves both the mother and the fetus as patients. During cesarean sections, the effectiveness and safety of electrocautery have also been evaluated; however, in these investigations, the mother was the only assessed patient [10,11]. The effects of the passage of high-frequency alternating electrical current through the fetus have not been adequately investigated. This study aimed to assess the impact of electrocautery on the fetus during cesarean section.

## 2. Materials and Methods

This retrospective study was conducted in Mersin University Hospital. All the patients who were delivered by Hakan Aytan (HA) and Huseyin Durukan (HD) with cesarean section between 1 January 2019 and 1 June 2023 and who met the inclusion criteria were enrolled. All the term pregnant women who had an elective obstetric indication (previous cesarean delivery, cephalopelvic disproportion, or abnormal fetal presentation) for cesarean delivery, whose newborns were delivered by HA and HD, and who gave informed consent were included. Exclusion criteria included preterm deliveries, pregnancies with anomalous or intrauterine dead fetuses, fetal distress or nonreactive nonstress tests, intrauterine growth restriction, preeclampsia/eclampsia, uncontrolled gestational diabetes, and placenta accreta spectrum disorders. Upon admission to our institution, patients were informed about the possibility of their data being used in retrospective studies, and written consent was obtained from those who agreed to participate. This study was approved by the institution’s ethics committee (number: 18.10.2023/682).

Both surgeons utilized a Pfannenstiel incision for skin access and a lower-segment transverse incision (Kerr) for uterine access. After skin incision with a scalpel, HA routinely used monopolar electrocautery (40 watts) in cut mode to divide subcutaneous abdominal tissues and fascia. He also used monopolar electrocautery (40 watts) in coagulation mode to achieve subcutaneous hemostasis. HD used a scalpel or scissors and did not use any electrocautery before delivering the fetus. All the other technical steps of the cesarean section were similar (double-layer uterine closure, first layer continuous locking, second layer continuous without locking sutures, closure of the visceral and parietal peritoneum, closure of the rectus fascia with nonlocking continuous sutures, subcutaneous tissue closure with single sutures, and skin closure with subepithelial continuous saturation). After the delivery of the fetus, delayed umbilical cord clamping was performed, and the blood samples were collected from the umbilical arteries after division of the umbilical cord. This sample was sent for blood gas analysis without delay (measured with an ABL800 FLEX blood gas analyzer).

The pediatrician received the newborn immediately after delivery. The initial examination was carried out in the delivery unit. The newborn was dried on an infant radiant warmer and stimulated; the head and neck were positioned to open the airway, and the secretions were cleared from the airway if needed. The 1st, 5th, and 10th minute APGAR scores were recorded by checking heart and respiratory rates, muscle tone, reflexes, and color. Oxygen saturation and the newborn’s body temperature were recorded. Tthe newborn was admitted to the intensive care unit if necessary.

When indicated, the newborns’ complete blood count tests and blood biochemistry analyses were carried out. The timing of these tests differed; however, they were performed within the first 6 h after the delivery.

Statistical analysis was accomplished with SPSS (Armonk, NY, USA: IBM, demo 22 version). The normality of the data was tested with the Kolmogorov–Smirnov test and histograms. The data were expressed as means ± standard deviations or medians (interquartile ranges) based on the distribution. Nominal data were expressed as numbers and percentages. A Chi-square test, *t*-test, or Mann–Whitney test was used for comparisons where appropriate. Correlation analysis was carried out with the Pearson correlation coefficient or Spearman’s rho tests. Linear regression analysis was performed with the parameters found to be significant in the correlation tests. Statistical significance was set at *p* < 0.05.

## 3. Results

A total of 1193 women had their newborns delivered via cesarean section by HA or HD during this period and 552 were eligible for this study; the monopolar electrocautery group consisted of 272 patients, and the no-electrocautery group consisted of 280. There were no maternal or fetal mortalities or severe morbidities. There were no burn cases due to electrocautery in any of the groups. The mean time from skin incision to birth of the fetus did not differ between the groups (3.36 ± 1.6 and 3.15 ± 1.8 min in the electrocautery and no-electrocautery groups, respectively (*p* = 0.612)). There were no differences with respect to total operation time (52.3 ± 10.2 min in the electrocautery group and 54.8 ± 12.4 min in the no-electrocautery group, *p* = 0.485) and change in pre- and postoperative hemoglobin values (1.18 ± 0.66 g/dL in electrocautery group and 1.32 ± 0.60 g/dL in the no-electrocautery group, *p* = 0.626). Comparisons of the demographic characteristics and the fetal 1st, 5th, and 10th minute Apgar scores, vital signs, and umbilical cord blood serum analytes between the groups are depicted in Table 1. The mothers in the electrocautery group were significantly older and gave birth to heavier newborns than the no-electrocautery group (32.6 ± 5.1 vs. 30.6 ± 6 years old, and 3372.9 ± 420.9 vs. 3299.9 ± 435.3 g, *p* < 0.001 and *p* = 0.045, respectively). The 1st and 5th minute Apgar scores were significantly higher in the electrocautery group (9 (1) vs. 8 (2) and 10 (1) vs. 9 (1), *p* = 0.001 and *p* = 0.024, respectively); however, this difference lost its significance at the 10th minute (10 (0) vs. 10 (0), *p* = 0.549) (Table 1). The median newborn pulse rate was significantly higher in the electrocautery group (148 (7) vs. 146 (6) beats per minute, *p* = 0.026) (Table 1). The umbilical cord blood pH and partial oxygen pressure were significantly higher in the electrocautery group compared to the no-electrocautery group (7.34 ± 0.06 vs. 7.31 ± 0.06, *p* < 0.001, and 25.5 (14.77) vs. 23 (16.08) mmHg, *p* = 0.025, respectively) (Table 1). The median umbilical cord blood serum calcium level was 1.51 (0.64 mmol/L in the electrocautery group, which was significantly lower than 1.9 (0.82) mmol/L in the no-electrocautery group (*p* = 0.002)). All the other cord blood serum parameters did not differ between the groups (Table 1).

The rate of newborn intensive care unit admission was 14.7% (*n*: 40) in the electrocautery group and 19.3% (*n*: 54) in the no-electrocautery group (Chi-square, *p* = 0.094). Ninety-four newborns (17%) were admitted to the intensive care unit. The most common indication was respiratory distress (10.7%), followed by hypoglycemia (4%), hyperbilirubinemia (2%), and thrombocytopenia (0.4%). The distribution of the newborn intensive care unit admission indications did not differ between the groups (*p* = 0.325). However, the incidence of hypoglycemia was significantly lower in the electrocautery group than in the no-electrocautery group (2.2% (*n*: 6) vs. 5.7% (n: 16), *p* = 0.035).

Comparisons of the newborn blood serum parameters are shown in Table 2. All of the assessed parameters were similar between the groups except potassium and total bilirubin levels. In the electrocautery group, the mean potassium level was lower and the total bilirubin level was higher compared to the no-electrocautery group (4.9 ± 0.6 vs. 5.2 ± 0.9 mEq/L, *p* = 0.017 and 9.3 ± 3.8 vs. 8.1 ± 3.9 mg/dL, *p* = 0.001, respectively) (Table 2).

The factors related to umbilical cord blood pH were assessed with correlation analysis (Table 3). Electrocautery usage, 1st and 5th minute APGAR scores, respiration rate, and cord blood pO_2_ and pCO_2_ were found to be significantly correlated with umbilical cord blood pH. Regression analysis was performed using these parameters to determine the independent predictors of umbilical cord blood pH (Table 3). With electrocautery usage, 1st minute APGAR scores, cord blood PO_2_, and PCO_2_ were independent predictors of fetal umbilical cord pH (Table 3).

Factors related to umbilical cord blood oxygen partial pressure (pO_2_) were analyzed with correlation analysis, and regression analysis was performed with the significantly correlated factors (Table 4). Umbilical cord blood pO_2_ significantly correlated with newborn pulse rate, usage of monopolar electrocautery, umbilical cord blood pH, partial carbon dioxide pressure (pCO_2_), and calcium levels (Table 4). Among these parameters, pH, pCO_2_, and calcium levels were independent predictors of umbilical cord blood oxygen partial pressure (Table 4).

Umbilical cord blood calcium levels were found to be significantly correlated with newborn body temperature, monopolar electrocautery usage, umbilical cord blood pO_2_, and potassium and sodium levels (Table 5). All of these parameters were found to be independent predictors of umbilical cord blood calcium levels during the regression analysis (Table 5).

## 4. Discussion

Energy devices have revolutionized surgical practice and have pioneered a new era of surgery. Today, these devices are present in almost every operation room, including those for gynecology and obstetrics. Cesarean section is a unique surgery in which the operator must consider the sake of two patients during the same operation. Electrocautery is as safe for the mother as any other operation performed on women [10]; however, interestingly, there are no studies that assess the fetal safety of this applied high-frequency electrical current that passes through the fetus and leaves the mother’s body through the dispersion pad. This study aimed to assess how safe monopolar electrocautery is for the fetus. The results show that the fetus may be affected by this passing electric current. The pulse rate increases, the 1st and 5th minute Apgar scores improve, the umbilical cord pH and pO_2_ increase, and the calcium level decreases. All of these changes are within normal limits and seem to have no clinical significance. The only finding that may have a clinical implication is hypoglycemia, which is seen less often in newborns whose mothers are subjected to electrocautery during cesarean section.

In monopolar electrocautery, high-frequency electrical current obtained from the electrosurgical unit flows from the active electrode through the tissues, including the fetus, during cesarean section. The current completes the circuit by leaving the body through the ground pad [2,12]. The fetus is delivered just after this current flow, and the abovementioned changes are observed in the fetus. All of these changes may be explained by assuming that this electric stimulation may trigger moderate acute stress in the fetus. As a response to this stress, an increased catecholamine release from the fetal adrenal glands may occur, increasing heart rate, Apgar scores, oxygen partial pressure, and umbilical cord pH and decreasing the risk of hypoglycemia.

The adrenal gland functions as a reservoir for catecholamines, which are subsequently released into the bloodstream through the activation of the sympathetic nervous system. The chromaffin cells located in the adrenal medulla serve as principal effectors of the sympathetic nervous system. Following splanchnic stimulation, the aforementioned cells undergo depolarization and generate action potentials, resulting in an influx of calcium ions through voltage-gated calcium channels [13]. The phenomenon of electric current flow is recognized to induce a transient physiological alteration in the affected organ or organism, which typically exhibits a reversible nature. This alteration can manifest as either inhibition or excitement [14]. According to reports, heightened sympathetic activity, which is evident during the acute stress reaction, induces chromaffin cells to exhibit a firing rate that is approximately 30 times greater [15,16].

Electric current from monopolar electrocautery may produce a temporary acute stress that stimulates the release of the stored catecholamines in the fetal adrenal medulla into the circulation. Released catecholamines may be responsible for the immediate physiologic changes detected just after delivery. The observed lower umbilical cord blood calcium levels may also be explained by an intracellular reflux of calcium during secretion from the gland. All these findings are within normal limits and are temporary, as the comparisons of the blood serum parameters measured after delivery do not differ. They do not seem to have clinical significance; however, they show that the predelivery electrocautery procedure affects the intrauterine fetus. This procedure can potentially affect the fetal umbilical cord pH and calcium levels directly.

The stimulatory effect of electrocautery on adrenal catecholamine release was reported before in a case of hemi-hepatectomy where the adrenal gland was stimulated by monopolar electrocautery, and this resulted in a hypertensive crisis in a patient with normal neuroendocrine function [17]. The release of catecholamines as a response to electrocautery has also been shown in animal models [18]. However, in these reports, there was direct trauma to the adrenal glands, and the response was severe hypertension, which is not the case in the present study. During surgery in patients with normal neuroendocrine function, electrocautery does not result in any hormonal changes if there is no direct intervention on a particular organ such as the adrenal glands. The fetus as a patient is different from an adult. The maturation of the central nervous system is incomplete. Fetal organ systems are more vulnerable, leading the fetus to be prone to distress with minimal stimuli [19]. The human fetal stress response in a 23-week-old human fetus needled in the hepatic (innervated) vein resulted in increased levels of cortisol and beta-endorphins as well as the “central sparing response” [20]. The researchers resolved this stress response by administering narcotics. Fetal stress as a result of exaggerated pain responses has been shown in eight-week-old infants, and fetal stress has been suggested as a contributor to preterm labor [21]. The fact that electrocautery is related to catecholamine release and that the fetus is prone to fetal stress with minimal adverse stimuli may be the underlying mechanism for the differences between the electrocautery group and the no-electrocautery group.

This study aimed to assess the safety of electrocautery regarding the fetus. We found significant differences that seem to be related to the usage of electrocautery. We assume the underlying mechanism may be the release of fetal catecholamines in response to the electric current; however, the catecholamine levels were not measured in the umbilical cord. This constitutes the main limitation of this retrospective study. This study’s strengths are that the operations were performed by the same two surgeons with the same electrocautery device, the same pediatricians assessed the newborns, the blood samples were analyzed using the same device, and the sample size was relatively large.

In conclusion, the results of this study show that monopolar electrocautery during cesarean section affects the fetus, but it is safe to use it. Although the physiologic changes are transient, are all within the normal ranges, and seem to have no clinical significance, electrocautery is independently associated with umbilical cord blood pH and calcium level. Electrocautery may be associated with a lower incidence of hypoglycemia. To clarify the underlying mechanism, prospective studies that analyze the catecholamines in the umbilical blood cord are needed.

## Figures and Tables

**Table 1:** Comparisons of the demographic characteristics and the newborn findings between the groups.

	Electrocautery Group (*n*: 272)	No-Electrocautery Group (*n*: 280)	*p*
Maternal age (years)	32.6 ± 5.1	30.6 ± 6	<0.001
Gestational week	38.3 ± 0.6	38.4 ± 0.9	0.105
Newborn birth weight (g)	3372.9 ± 420.9	3299.9 ± 435.3	0.045
Gravidity	2 (2)	2 (3)	0.078
Parity	1 (2)	1 (2)	0.006
APGAR 1st minute	9 (1)	8 (2)	0.001
APGAR 5th minute	10 (1)	9 (1)	0.024
APGAR 10th minute	10 (0)	10 (0)	0.549
Newborn pulse (per min)	148 (7)	146 (6)	0.026
Newborn saturation	99 (2)	98 (1)	0.085
Newborn temperature (°C)	36.4 (0.4)	36.5 (0.5)	0.646
Newborn respiration rate (per min)	46 (5)	46 (5)	0.864
Cord blood pH	7.34 ± 0.06	7.31 ± 0.06	<0.001
Cord blood pO_2_ (mmHg)	25.5 (14.77)	23 (16.08)	0.025
Cord blood pCO_2_ (mmHg)	46 (10.18)	46.1 (8.53)	0.508
Cord blood potassium (mmol/L)	4.9 (0.8)	4.9 (0.5)	0.483
Cord blood sodium (mmol/L)	135 (5)	135 (10)	0.582
Cord blood calcium (mmol/L)	1.51 (0.64)	1.9 (0.82)	0.002

**Table 2:** Comparisons of newborn serum parameters.

	Electrocautery Group	No-Electrocautery Group	*p*
Hemoglobin (g/dL)	18.7 ± 2.7	19.5 ± 2.2	0.052
Hematocrit (%)	52.6 ± 8.2	54.9 ± 6.4	0.059
WBCs	13,533.9 ± 5504.4	13,876.5 ± 5678.9	0.704
Platelets	276,500 (93,750)	274,500 (92,750)	0.900
Blood glucose (mg/dL)	73.3 ± 16.8	70.2 ± 14.8	0.286
Urea (mg/dL)	16.77 (10.1)	19 (8.4)	0.501
Creatinine (mg/dL)	0.57 (0.25)	0.6 (0.18)	0.249
ALT (U/L)	15 (9.5)	17.5 (9.53)	0.324
AST (U/L)	49.5 (23.5)	53.5 (36)	0.107
Sodium (mEq/L)	138.4 ± 3.38	138.7 ± 4.3	0.688
Potassium (mEq/L)	4.9 ± 0.6	5.2 ± 0.9	0.017
Calcium (mEq/L)	9.4 ± 0.8	9.5 ± 1.6	0.611
Direct bilirubin (mg/dL)	0.59 ± 0.1	0.59 ± 0.2	0.712
Total bilirubin (mg/dL)	9.3 ± 3.8	8.1 ± 3.9	0.001
Albumin (g/L)	3.6 ± 0.4	3.5 ± 0.4	0.721
Total protein (g/L)	5.4 ± 0.6	5.4 ± 0.6	0.936

WBC: White blood cell, ALT: Alanine aminotransferase, AST: Aspartate aminotransferase.

**Table 3:** Correlation and regression analyses of the factors related to umbilical cord pH.

	Correlation Analysis		Regression Analysis		
	r	*p*	beta	*p*	95% CI
Electrocautery usage	−0.208	<0.001	−0.164	<0.001	−0.03–−0.011
Newborn respiration rate	−0.093	0.03	−0.057	0.122	−0.002–0
APGAR 1st minute	0.256	<0.001	0.184	0.003	0.003–0.014
APGAR 5th minute	0.239	<0.001	0.031	0.619	−0.006–0.011
Umbilical cord blood pO_2_	0.197	<0.001	0.107	0.005	0–0.001
Umbilical cord blood pCO_2_	−0.414	<0.001	−0.359	<0.001	−0.003–0.002

pO_2_: partial oxygen pressure, pCO_2_: partial carbon dioxide pressure.

**Table 4:** Correlation of umbilical cord blood oxygen partial pressure with other parameters.

	Correlation Analysis		Regression Analysis		
	r	*p*	beta	*p*	95% CI
Newborn pulse rate (per minute)	0.086	0.044	0.064	0.121	−0.03–0.28
Electrocautery usage	−0.095	0.025	−0.076	0.07	−3.11–0.12
Umbilical cord blood pH	0.195	<0.001	0.097	0.035	1.09–30.32
Umbilical cord blood pCO_2_	−0.170	<0.001	−0.171	<0.001	−0.29–−0.09
Umbilical cord blood calcium	0.142	0.001	0.172	<0.001	1.44–3.98

pCO_2_: partial carbon dioxide pressure.

**Table 5:** Correlation and regression analyses of umbilical cord blood calcium level with other parameters.

	Correlation Analysis		Regression Analysis		
	r	*p*	beta	*p*	95% CI
Newborn body temperature	0.084	0.048	0.119	0.003	0.06–0.29
Electrocautery usage	0.134	0.002	0.098	0.014	0.03–0.22
Umbilical cord blood pO_2_	0.142	0.001	0.097	<0.001	0.01–0.02
Umbilical cord blood potassium	0.294	<0.001	−0.171	<0.001	0.14–0.29
Umbilical cord blood sodium	0.248	<0.001	0.172	<0.001	0.01–0.03

pO_2_: partial oxygen pressure.

## Data Availability

The datasets analyzed during the current study are available from the corresponding author.
